# Sex-related DNA methylation differences in B cell chronic lymphocytic leukemia

**DOI:** 10.1186/s13293-018-0213-7

**Published:** 2019-01-07

**Authors:** Shuchun Lin, Yun Liu, Lynn R. Goldin, Chen Lyu, Xiangyin Kong, Yan Zhang, Neil E. Caporaso, Song Xiang, Ying Gao

**Affiliations:** 1 Shanghai Institutes for Biological Sciences, University of Chinese Academy of Sciences, Chinese Academy of Sciences, Shanghai, China; 2 The MOE Key Laboratory of Metabolism and Molecular Medicine, Department of Biochemistry and Molecular Biology, School of Basic Medical Sciences, Fudan University, Shanghai, China; 3 Division of Cancer Epidemiology and Genetics, National Cancer Institute, National Institutes of Health, Bethesda, MD USA; 4 Department of Epidemiology and Biostatistics, School of Public Health-Bloomington, Indiana University, Bloomington, IN USA; 5 College of Life Sciences and Oceanography, Shenzhen University, Shenzhen, Guangdong Province China

**Keywords:** DNA methylation, Chronic lymphocytic leukemia, B cell, Sex, EWAS

## Abstract

**Background:**

Men are at higher risk of developing chronic lymphocytic leukemia (CLL) than women. DNA methylation has been shown to play important roles in a number of cancers. There are differences in the DNA methylation pattern between men and women. In this study, we investigated whether this contributes to the sex-related difference of B cell CLL risk.

**Methods:**

Using the HumanMethylation450 BeadChip, we profiled the genome-wide DNA methylation pattern of CD19^+^ B cells from 48 CLL patients (29 female patients and 19 male patients) and 28 healthy people (19 women and 9 men).

**Results:**

We identified 1043 sex-related differentially methylated positions (DMPs) related to CLL, 56 of which are located on autosomes and 987 on the X chromosome. Using published B cell RNA-sequencing data, we found 18 genes covered by the DMPs also have different expression levels in male and female CLL patients. Among them, *TRIB1*, an autosome gene, has been shown to promote tumor growth by suppressing apoptosis.

**Conclusions:**

Our study represents the first epigenome-wide association study (EWAS) that investigates the sex-related differences in cancer, and indicated that DNA methylation differences might contribute to the sex-related difference in CLL risk.

**Electronic supplementary material:**

The online version of this article (10.1186/s13293-018-0213-7) contains supplementary material, which is available to authorized users.

## Background

Chronic lymphocytic leukemia (CLL) is characterized by proliferation and accumulation of malignant B lymphocytes in the peripheral lymphoid tissues and bone marrow. It is one of the most common leukemias among adults in the western world [1]. Its occurrence in men and women is drastically different [2]. For instance, the Surveillance, Epidemiology, and End Results (SEER) database indicated that in 1975–2001, the US CLL incidence per 100,000 per year was 5.0 for men and 2.5 for women [3, 4]. In addition, female CLL patients have better 10-year survival rates and show better response to treatment [5]. Understanding the mechanism behind these sex-related differences will provide valuable insights into CLL.

DNA methylation plays important roles in regulating gene expression. There are considerable differences in the DNA methylation pattern between men and women. For instance, recent studies on human blood DNA revealed significant sex-related differences in its methylation pattern [6–8]. DNA methylation changes are linked to many diseases [9]. In CLL patients, a strong change in DNA methylation pattern is reported [10]. This suggests that DNA methylation could play a role in the sex-related differences in CLL. However, to date, solid evidence is lacking.

We report here an epigenome-wide association study (EWAS) of CLL. Our study revealed 1043 sex-related differentially methylated positions (DMPs) in CLL. Using available RNA-sequencing data, we found 18 sex-related differentially expressed genes (DEGs) that overlapped with these DMPs. A number of these genes have been reported to be associated with aggressive CLL progression. To our knowledge, this study is the first EWAS that investigates the sex-related differences in cancer. The differently methylated/expressed genes we identified could be potential markers for CLL risk assessment and drug targets for CLL treatment.

## Methods

### Sample preparation

In this study, 48 CLL subjects and 28 unrelated healthy controls were recruited from the NCI CLL Registry [11]. A total of 92 blood samples were collected, with multiple samples collected from 8 subjects. Then, B lymphocytes were selected from cryopreserved peripheral blood lymphocytes using a CD19 antibody. Cell purity was evaluated with flow cytometry using propidium iodide and CD45/CD19 antigens. Samples with greater than 90% purity were processed for DNA extraction and methylation analysis.

### Datasets for DMP replication

To replicate the DMPs we detected, we requested two DNA methylation datasets accessed by 450K from The European Genome-phenome Archive (EGA), EGAD00010000254 [12] and EGAD00010000871 [13]. Both contain B cell samples from CLL patients and healthy people (Additional file 1: Table S1).

### Datasets for DEG analysis

We requested two RNA-sequencing datasets for B cell from CLL patients: EGAD00001000258 [14] from EGA and GSE66117 [15] from Gene Expression Omnibus (GEO). Sex information of GSE66117 was obtained from the author. Data for healthy immortalized B cells (GSE16921 [16]) was used as a control. Since the mRNA expression of immortalized B cells might differ from normal B cells, two additional control datasets were requested. One contains two collections of CD19^+^ B cells from healthy women (GSM1523501 and GSM1523502 from GSE62246 [17]); the other contains five collections of CD19^+^ B cells from healthy men (GSM1820115, GSM1820116, GSM1820117, GSM1820118, and GSM1820119 from GSE70830). Sex information was obtained from the author.

### DMP analysis

**Fig. 1:**
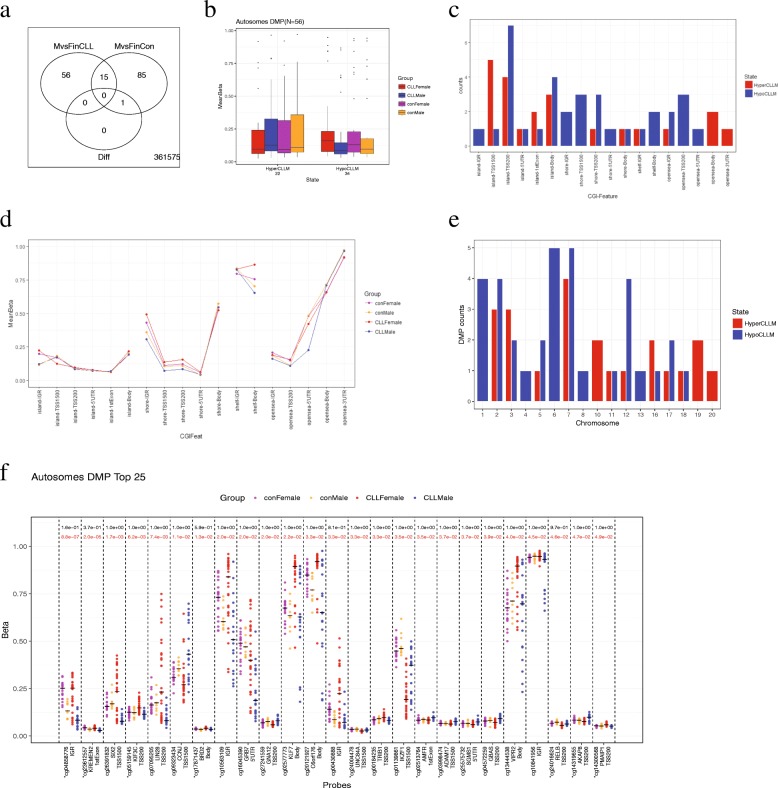
**a** MvsFinCLL: significant DNA methylation difference probes between male and female CLL patients; MvsFinCon: significant DNA methylation difference probes between healthy men and women; Diff: significant interaction term. **b** Mean *β* of each probe in each group was used. **c** Autosomal DMPs stratified by gene features (TSS200, TSS1500, 1stExon, 5′UTR, Body, IGR) and CpG features (Island, Shore, Shelf, Open Sea). **d** Each dot represented the mean *β* value of DMPs. **e** Autosomal DMPs stratified by autosomes. **f** Top 25 autosomal DMPs. Black bar indicated the median *β* value of each group. Black number in each probe above indicated *q* value in controls, and red number indicated *q* value in CLL. Probes marked with an asterisk indicated the replicated DMPs

**Fig. 2:**
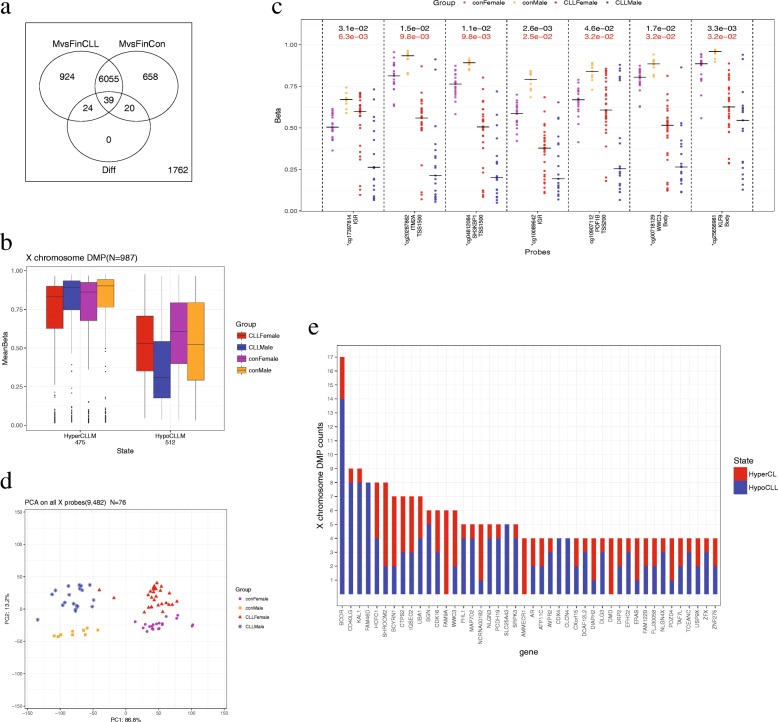
**a** X chromosomal DMPs were counted as 924 + 24 + 39 = 987. **b** X chromosomal DMPs stratified by hyper- and hypo- DMP. **c** The 7 opposite X chromosomal DMPs. **d** PCA analysis according to all samples’ *M* value in all 450K X chromosomal probes (*N* = 9482). **e** Genes with X chromosomal DMPs over 4.

After passing quality control of 450K BeadChip, our dataset contains 361,732 autosomal probes and 9482 X chromosomal probes from 89 samples. Samples obtained after the first diagnosis of CLL (*N* = 76) were next used to identify DMP. Autosomal DMPs and X chromosomal DMPs were detected separately. We used linear regression following the R package *limma* [18] to detect probes with significant DNA methylation differences between male and female CLL patients and between healthy men and women, along with an interaction term. Age was adjusted in the model. *P* values were corrected for multiple testing by Benjamini-Hochberg FDR (*q* value). Probes with FDR under a threshold of 0.05 (*q* value < 0.05) were considered significant. For the autosomal probes, 71 were significant between male and female CLL patients, and 101 were significant between healthy men and women. These 2 groups shared 15 common probes, which had the same methylation difference direction, but showed no difference in the interaction term. Autosomal probes that had significant methylation differences between male and female CLL patients, but not between healthy men and women, were defined as sex-related autosomal DMPs (*N* = 56, Fig. 1a). For the X chromosomal probes, 7042 were significant between male CLL and female CLL patients, and 6772 were significant between healthy men and women. These 2 groups shared 6094 common probes, 39 of which were significant in the interaction term. X chromosomal probes that had significant methylation differences between male and female CLL patients, but not between healthy men and women (*N* = 948), as well as those significant in the interaction term (*N* = 39), were defined as X chromosomal DMPs (Fig. 2a). The same method was applied to the datasets for DMP replication. Details of the interaction term were in Additional file 2.

### DEG analysis

DEGs were identified by the *limma* package [19] using an interaction linear model adjusted for the study batch. *P* values corrected by FDR under the cutoff of 0.05 (*q* value < 0.05) were considered significant. Sex-related DEGs were defined as genes with significantly different expression levels between male and female CLL patients, but not between healthy men and women.

### Other analyses

Methods for analysis of 450K BeadChip data, differentially methylated region (DMR), functional epigenetic module (FEM), and Gene Ontology (GO), are shown in Additional file 2.

## Results

### Autosomal DMPs

The characteristics of subjects are summarized in Table 1. We identified 56 DMPs among 450k autosomal CpG sites, which had significant methylation differences between male and female CLL patients, but not between healthy men and women (Fig. 1a). Among them, 22 were hypermethylated (hyper-DMPs) and 34 were hypomethylated (hypo-DMPs) in male CLL patients, compared to female patients (Fig. 1b). Both hypo-DMPs and hyper-DMPs were mainly enriched in CpG islands and promoter regions (Fig. 1c, both *p* values < 0.01 in Fisher’s test). These 56 autosomal DMPs showed little difference in DNA methylation between healthy men and women (Fig. 1d). They are enriched in chromosomes 2, 5, 6, and 7 (Fig. 1e, all *p* values < 0.05 in Fisher’s test). Noticeably, most hypo-DMPs were located in chromosome 6, a chromosome known to be associated with human immune diseases [20]. These 56 DMPs were located in 48 genes. Gene Ontology (GO) analysis revealed that these genes are mainly involved in metabolic processes, leukocyte and lymphocyte homeostasis, activation and differentiation, and protein and nucleic acid binding, and are located in the cell plasma and at cell junctions (Additional file 3).

**Table 1:** Demographic character of the 76 samples

		Female	Male	*p* value
Numbers	CLL	29	19	0.69^a^
	Control	19	9	
Age	CLL	63.28 (14.83)	61.21 (8.55)	0.56^b^
	Control	58.26 (10.61)	47.22 (18.43)	0.13^b^

^a^In chi-squared test

^b^In Wilcoxon rank sum test. Age was shown in the value of mean (sd)

The top 25 DMPs ranker by Δβ and *q* value are shown in Fig. 1f. Probe cg16045390, the top one, is located in the 5′UTR enhancer region of gene *GRB7*. It is a hypo-DMP. Increased *GRB7* expression has been reported to be related to the late stage of CLL [21]. This CpG site in male CLL patients is also significantly hypomethylated compared to both healthy men and women. Another top DMP, cg24016624, is a hyper-DMP and is located in TSS200 of gene *RELB*. In the apoptosis-resistant B cells, gene *RELB* was found to be inhibited in male CLL patients but upregulated in female CLL patients [22]. Hyper-DMP cg01139861 is located in TSS1500 of gene *IKZF1*, which is a B-lymphoid transcription factor and is essential for early B cell development. IKZF1 represses myeloid differentiation by limiting leukemic transformation of pre-B cells [23].

### X chromosomal DMPs

A total of 987 CLL sex-related DMPs were identified in the X chromosome (Fig. 2a). These included probes that had significant methylation differences between male and female CLL patients but not between healthy men and women (*N* = 948) and probes that were significant in the interaction term (*N* = 39). These DMPs were mainly enriched in promoter, gene body, and the island regions (Additional file 1: Figure S1, all *p* values < 0.01). Large differences in DNA methylation between male and female CLL patients were observed, but the difference was less prominent between healthy men and women (Fig. 2b). The DNA methylation differences between male and female CLL patients and between healthy men and women were completely opposite for 7 DMPs in the interaction term (Fig. 2c). If female CLL patients and healthy women were compared, probe cg17397814 had increased methylation, whereas if male CLL patients and healthy men were compared, it had decreased methylation. No other DMPs were found to possess this property.

Since more DMPs were identified in the X chromosome than in autosomes, we conducted a principal component analysis (PCA) using the methylation values of all 450K X chromosomal probes. The result showed that the first two PCs could classify all the 76 samples into four groups according to sex and disease status (Figure 2d). This indicated that the global DNA methylation status in the X chromosome was drastically different between male and female CLL patients.

**Fig. 3:**
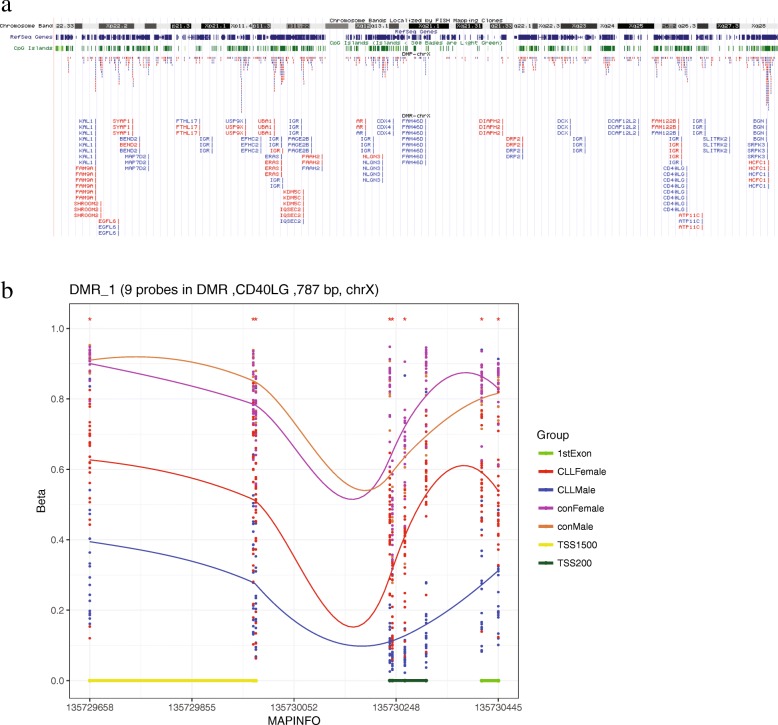
**a** Coordinate of the X chromosomal DMPs and DMRs. Hyper-DMPs were shown in red, and hypo-DMPs were shown in blue. **b** A DMR in gene *CD40LG*. DMPs were marked with an asterisk above, and all could be replicated

The 987 X chromosomal DMPs are located in 407 genes. GO analysis revealed that they are mainly involved in cellular component organization, cell-cell signaling, and receptor binding, and are located in lytic vacuoles and at cell junctions (Additional file 3). There were 44 genes covering at least 4 DMPs (Fig. 2e). All DMPs in genes *FAM9A*, *AMMECR1*, and *DMD* were hyper-DMPs. Interestingly, we found that a number of DMPs were located in Xq28 (Fig. 3a), a region known to be associated with the PAR2 pseudoautosomal regions, where genes are inherited like autosomal genes [24].

Thirty-eight differentially methylated regions (DMRs) were identified in the X chromosome (Additional file 3). All DMPs in 6 DMRs, located in gene *FAM9A*, *UBA1*, *DIAPH2*, *SHROOM2*, *KDM5C*, and *SYAP1*, are hyper-DMPs (Fig. 3a). The top DMR (Stouffer FDR = 1.16e−43) is located in gene *CD40LG*. It covers 8 hypo-DMPs that are all located in the promoter region (Fig. 3b). CD40LG promotes B cell maturation by engaging CD40 on the B cell surface [25]. Using mouse embryonic fibroblasts cell lines transfected with *CD40LG* to mimic the CLL lymph node and vascular microenvironments, Hamilton et al. found that the survival and proliferation of peripheral blood mononuclear cells from CLL patients were markedly enhanced [26]. However, *CD40LG* was not identified as a CLL-related DEG in our study; its role in CLL requires further study.

### DMP replication

The datasets for DMP replication include B cell DNA methylation data for 116 female CLL patients, 186 male CLL patients, 9 healthy women, and 12 healthy men (Additional file 1: Table S1). Using the same method applied to our data, we could reproduce 36 autosomal DMPs (Additional file 1: Figure S2a), and 732 X chromosomal DMPs identified in our data (Additional file 3). Six out of the 7 X chromosomal DMPs that had reversed DNA methylation changes if CLL patients and healthy controls were compared (Fig. 2c) could be reproduced with this data (Additional file 1: Figure S2b). Twenty-three out of 44 genes with at least 4 DMPs identified in our data were reproduced with this data (Additional file 1: Figure S2c). All DMPs located in genes *CD40LG*, *NCRNA00182*, *NLGN3*, *DLG3*, *FAM122B*, *USP9X ZFX*, and *AMMECR1* were reproduced with this data. All DMPs of 13 DMRs in genes *CD40LG*, *PAGE2B*, *NLGN3*, *FAM122B*, *BGN*, *SRPK3*, *MAP7D2*, *SHROOM2*, *KDM5C*, *SYAP1*, *USP9X*, and 2 IGR (DMR_29, DMR_35) were reproduced. A full list of the replicated DMPs is shown in Additional file 3.

### DEG analysis

Public RNA-Seq data of B cells from 50 female CLL patients, 84 male CLL patients, 17 healthy women, and 24 healthy men (Additional file 1: Table S1) were retrieved to test whether the DMPs we detected were linked to gene expression changes. With this data, we detected 83 sex-related DEGs, including 59 autosomal genes and 24 X chromosomal genes (Additional file 1: Figure S3a). Combining this result with our data, we identified 18 genes with significant differences in both DNA methylation and gene expression between male and female CLL patients (DNAm-DEGs). These 18 DNAm-DEGs cover 48 DMPs, of which 35 (from 15 DNAm-DEGs) were reproduced with the The European Genome-phenome Archive (EGA) data (Additional file 1: Figure S3b). The top DNAm-DEG, *MAP7D2* (log_2_FC = − 4.7, *q* value = 2.3e−17), covers 5 DMPs in a single DMR.

**Fig. 4:**
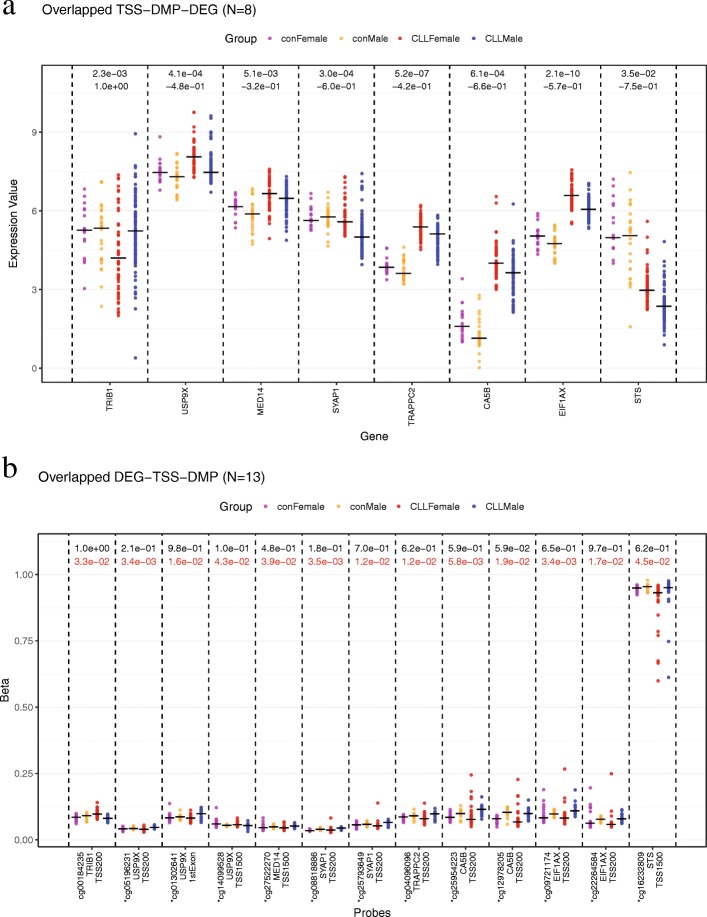
**a** The 8 DEGs that showed a negative correlation between expression and DNA methylation in the promoter regions. Expression value was shown in log_2_CPM scale, black number in each gene above indicated *q* value in CLL and controls respectively. **b** The 13 DMPs included in **a**

DNA methylation in the promoter region is known to be negatively correlated with gene expression. We further restricted our analysis to DMPs in the promoter regions. This revealed that the expression of 8 DNAm-DEGs, *TRIB1*, *USP9X*, *MED14*, *SYAP1*, *TRAPPC2*, *CA5B*, *EIF1AX*, and *STS* (Fig. 4a), was negatively correlated with methylation status of all their DMPs in the promoter regions (TSS-DNAm-DEGs, the 13 mapped DMPs are shown in Fig. 4b). Among these, *TRIB1* is probably the most interesting. It is located in chromosome 8 and has one hypo-DMP in TSS200. Its mRNA abundance in male CLL patients was about two times higher than that in female patients. High expression of *TRIB1* has been shown to activate the NFκB pathway, which suppresses apoptosis, and leads to a clinically more aggressive tumor phenotype [27]. Thus, increased *TRIB1* level could contribute to the more serious disease state in male CLL patients.

In the above analysis, data for immortal B cell was used as a control. Its mRNA expression may be different from normal B cells. To address this problem, we requested RNA-Seq data of normal B cell from 5 healthy men and 2 healthy women from Gene Expression Omnibus (GEO). Analysis of these 7 samples showed that the expression of 11 DNAm-DEGs we identified was not significantly different between healthy men and women (Additional file 3). As the relatively small sample size could introduce artifacts into our analysis, we further compared our results to a study that evaluated gene expression differences in B cells between men and women by microarray [28]. This analysis indicated that none of the DNAm-DEGs we identified had significantly different expression levels in the B cells from healthy men and women in their available data. Therefore, in our analysis, data for immortal B cell as a control did not substantially affect our results. A full list of DEGs is shown in Additional file 3.

## Discussion

According to the gene mutation status of the immunoglobulin heavy-chain variable (IGHV), CLL patients can be separated into 2 prognostic subgroups. Patients with mutated IGHV genes (M-CLL) have better outcome compared with those unmutated (U-CLL) [29]. Reports have shown that the subgroups of CLL have distinct methylation patterns [12, 30, 31]. Kulis et al. [12] identified 3265 CpGs of 450K that were differentially methylated between U-CLL and M-CLL. Based on Kulis et al., Queiros et al. [31] used a support vector machine (SVM) model with 5 CpGs of 450K to classily CLL into 3 subgroups, including M-CLL, U-CLL, and I-CLL (a group that showed an intermediate DNA methylation pattern between U-CLL and M-CLL). To detect the impact of CLL subgroups on our study, we first downloaded the list of 3265 CpGs from Kulis et al. Compared to our DMPs with this list, we found that only 2 CpGs were overlapped (cg15325759 and cg00868980, all were X chromosomal DMPs). This suggested that CLL subgroups should have little impact on our study. We next applied the same SVM model from Queiros et al. to classify our CLL samples (Additional file 1: Table S2). Results of this analysis showed that the distribution of CLL subgroups was not significant between male and female CLL patients (*p* value = 0.92, in chi-squared test). This indicated that the DMPs we found should not be caused by the distribution bias of CLL subgroups between male and female patients. Based on this classification, we applied the ANOVA model to test whether our DMPs were associated with CLL subgroups. With the cutoff of FDR adjusted *p* (*q* value) < 0.05, only 7 DMPs showed significant within 3 CLL subgroups (all were X chromosomal DMPs, Additional file 3). Thus, we considered the CLL subgroups should have little impact on our results.

Studies showed that the origin and the differential of B cells could affect the DNA methylation of CLL [12, 32]. Kulis et al. [12] found that B cells had different methylation patterns within their subtypes, which included CD19+ B cells, NBC (native B cells), CD5+ NBC, csMBC (class-switched-memory B cells), and ncsMBC (non-class-switched-memory B cells). They also suggested that U-CLL might derive from nongerminal center experienced cells (e.g., CD5+, CD27- B cells), while M-CLL from germinal center experienced cells (e.g., CD27+ B cells). Oakes et al. [32] found that CLL could maintain some epigenetic imprints from their B cell origin. To study the impact of B cell origin on this study, we requested the normal B cells samples from Kulis et al. [12], including 5 subtypes of B cells (Additional file 1: Table S3). With this data, we could detect the CpGs that showed significant methylated difference within these 5 subtypes. ANOVA model was applied to this analysis. CpG that had *q* value < 0.05 and |standard deviation of β among 5 groups| > 0.1 was considered differentially methylated within these 5 subtypes (Additional file 3). Finally, we could compare our DMPs to the CpGs we detected associated with B cells subtypes. We found that 702 (70.1%) X chromosomal DMPs and 52 (92.9%) autosomal DMPs were not included in the CpGs associated with B cell subtypes. This analysis indicated that most of our DMPs should not be involved in the B cell differentiation.

Many genes are silenced on one of the X chromosomes in female mammals due to X chromosome inactivation (XCI) [33]. Studies suggest that about 15% of genes may escape from XCI and an additional 10% are expressed at variable levels [34, 35]. A number of genes were heterogeneous in their X chromosome inactive status. In some individuals, they escape from XCI, and in some, they do not [36]. DNA methylation is known to play a key role in XCI [37]. Studies have shown that CpG islands have a tendency to be methylated on the inactive X chromosome and unmethylated on the active X chromosome, whereas the CpG islands of genes escaping XCI often remain unmethylated on both X chromosomes [38]. The 450K array should detect DNA methylation in both X chromosomes of the female subjects, and it is very likely that some of the 987 X chromosomal DMPs (covering 407 genes) we identified were subject to XCI. Therefore, it is possible that there were more X chromosomal DMPs than autosomes DMPs because of XCI and the false positive rate should not be the same between autosomal and X chromosomal DMPs. To minimize this false positive rate, we analyzed the autosomes and X chromosome separately. Noticeably, most X chromosomal DMPs showed no methylation difference between healthy men and women, except for the 39 DMPs in the interaction term. This indicated that most X chromosomal DMPs we identified were not due to XCI, but caused by sex-related differences of CLL.

To further explore the XCI escape status of these 407 X chromosomal DMP-covered genes we detected, we compared our results with previously published studies. Two studies used RNA expression and single nucleotide polymorphism (SNP) data to classify X chromosomal genes into categories as always, never, or sometimes (heterogeneous) escape XCI [35, 39]. Zhang et al. used immortalized B cells [39, 40], and 221 genes they identified overlapped with our X chromosomal DMP-covered genes, of which 62.9% could always or sometimes escape XCI. Cotton et al. used fibroblast and lymphoblastoid cell lines [35], and 279 genes they identified overlapped with our X chromosomal DMP-covered genes, of which 47.3% could always or sometimes escape XCI. The study by Zhang et al. is more relevant to our study, since it used an immortalized B cell line that was closer to the human biospecimen we used. Combining their data with ours, we found that all of the X chromosomal DNAm-DEGs we identified could always or sometimes escape XCI (Table 2). A third study by Moen et al. classified CpG sites that escaped methylation on the inactive X chromosome [41]. Eleven of the DNAm-DEGs we identified (*USP9X*, *TCEANC*, *DDX3X*, *CDK16*, *MED14*, *ZRSR2*, *EIF1AX*, *SYAP1*, *TRAPPC2*, *CXorf38*, and *RIBC1*) were covered by the CpG sites they identified. In addition, 475 (48.1%) of the X chromosomal DMPs we identified were hyper-DMPs. This percentage is higher than expected. Altogether, this suggested that most of the X chromosomal DNAm-DEGs we identified could escape XCI.

**Table 2:** Overlapped X chromosomal DMP-covered genes in two XCI studies

	Zhang et al.			Cotton et al.		
	Always escape	Heterogeneous	Always inactive	Always escape	Heterogeneous	Always inactive
X chromosomal DMP-covered genes (*n* = 407)	22	117	82	39	93	147
DMR-covered genes (*n* = 31)	*USP9X*; *UBA1*; *KDM5C*; *HCFC1*	*SYAP1*; *MAP7D2*; *FAAH2*; *DIAPH2*; *FAM122B*; *ATP11C*	*NLGN3*; *DRP2*; *BGN*	*SYAP1*; *UBA1*	*KAL1*; *SHROOM2*; *BEND2*; *MAP7D2*; *USP9X*; *EFHC2*; *KDM5C*; *IQSEC2*; *CD40LG*; *BGN*; *HCFC1*	*EGFL6*; *FAAH2*; *AR*; *NLGN3*; *DIAPH2*; *DRP2*; *DCX*; *ATP11C*
X chromosomal DNAm-DEGs (*n* = 17)	*USP9X*; *DDX3X*; *CDK16*; *MED14*; *EIF1AX*; *TRAPPC2*; *STS*	*ZNF275*; *ERCC6L*; *TBC1D25*; *ZRSR2*; *MAP7D2*; *SYAP1*; *CA5B*; *CXorf38*	NA	*DDX3X*; *ZRSR2*; *SYAP1*; *CA5B*; *CXorf38*; *STS*	*USP9X*; *TCEANC*; *CDK16*; *MED14*; *MAP7D2*; *EIF1AX*; *TRAPPC2*; *RIBC1*	*ZNF275*; *TBC1D25*

In addition, if we considered X chromosomal DMPs with its median *β* value over 0.8 or under 0.2 in female CLL patients as totally methylated or totally unmethylated on both X chromosome, 549 X chromosomal DMPs were identified (Additional file 1: Figure S4a). The methylation of these DMPs was in binomial distribution, same as the autosomal DMPs. These 549 DMPs were located in 270 genes, 43 of which had at least 3 DMPs (Additional file 1: Figure S4b). These 270 genes included 26 DMRs we detected before, and all the X chromosomal DNAm-DEGs with the exception of *ERCC6L*.

Dunford et al. suggested that tumor suppressor genes escape from XCI could protect females from complete functional loss by a single mutation, which contributes to the reduced cancer incidence in females across a variety of tumor types [42]. Among the 6 genes they detected, 5 coincide with the X chromosomal DMP-covered genes we identified (*ATRX*, *KDM5C*, *KDM6A*, *MAGEC3*, and a DNAm-DEG, *DDX3X*). Genes *KDM5C*, *KDM6A*, *MAGEC3*, and *DDX3X* had at least 1 DMP hypomethylated in female CLL patients, and *DDX3X* was also over-expressed in female CLL patients. These 5 genes likely play a role in the sex-related difference in CLL risk.

X chromosomal DEGs could interact with autosomal genes and affect their function. We used the functional epigenetic module (FEM) algorithm to detect such interactions. FEM seeks modules of functionally related genes that exhibit differential promoter DNA methylation and differential expression by using protein-protein interaction network, assuming an inverse association between promoter DNA methylation and gene expression [43]. We found 1 of the X chromosomal TSS-DNAm-DEGs, *MED14*, was a hotspot (Additional file 1: Figure S5). It interacts with 89 genes, 87 of them were autosomal (Additional file 3). *MED14* was also included in 1 of our GO enrichment terms, receptor binding. Therefore, DNA methylation change in the *MED14* promoter could not only regulate its own expression, but also the expression of a number of autosomal genes.

In addition to the X chromosomal DMPs, our study also identified 56 autosomal DMPs. Our FEM analysis did not reveal any interaction between the autosomal DMP-covered genes and the X chromosomal DEGs; what causes the DNA methylation difference between male and female CLL patients requires further study. Among the autosomal DMP-covered genes, *TRIB1* was identified as a TSS-DNAm-DEG and probably plays an important role in CLL through its function in the NFκB pathway and apoptosis. Genes *GRB7*, *RELB*, and *IKZF1* contain a single DMP each. The functions of these genes are related to CLL, and DNA methylation changes of them are associated with more severe CLL prognosis in men.

## Conclusions

Our study revealed a connection between the sex-related differences in DNA methylation and the CLL disease risk and outcome. A large number of X chromosomal sex-related DMPs were identified, and our data suggests that this is mainly contributed by XCI escape of many X chromosomal genes in female CLL patients. A number of the autosomal and X chromosomal DMPs we identified are located in genes with important functions in CLL-related cellular processes, suggesting that these genes likely contribute to the difference of CLL risk between sexes. In addition to these mechanistic insights, the large number of DMPs we identified and the related genes could be potential biomarkers for CLL risk and prognosis and potential drug targets.

### Perspectives and significance

Our study represents the first EWAS that investigates the sex-related differences in cancer and implicates that DNA methylation plays a role in the sex difference of CLL risk. We identified 1043 sex-related differentially methylated positions. Among them, DNA methylation alterations in *GRB7*, *RELB*, *IKZF1*, and *CD40LG*, genes associated with aggressive CLL progression, were found in male patients. We also found hypomethylation of *TRIB1* in male patients along with over-expression, a gene that promotes tumor growth by suppressing apoptosis. In addition, to provide insights into the sex bias of CLL risk, our study also identified potential targets for CLL treatment.

## Additional files

Additional file 1: **Table S1.** Samples for RNA-Seq analysis and replication of DNA methylation. **Table S2.** CLL subgroups of this study classified by Queiros et al. **Table S3.** Normal B cells subtypes samples from Kulis et al. **Figure S1.** Sex-related X chromosomal DMPs stratified by gene features and CpG features. **Figure S2a.** Thirty-six replicated autosomal DMPs. b. The 7 X chromosomal DMPs of figure 2d in EGA data. c. Genes with numbers of X chromosomal DMPs ≥ 4 in EGA data. **Figure S3a.** CLL sex-related DEGs. b. The 18 DNAm-DEGs and their DMPs. **Figure S4a.** There were 549 X chromosomal DMPs with median β < 0.2 or median β > 0.8 in CLL females. b. Genes with at least 3 DMPs in Figure S4a (*N*=43). **Figure S5.** *MED14* hotspot detected by FEM algorithm. (DOCX 3039 kb)

Additional file 2: Supplemental methods. (DOC 93 kb)

Additional file 3: Results detail of CLL sex-related DMPs, DMRs, DEGs, GO, and FEM. (XLSX 4427 kb)
