# Supplementary material for: Sex-related DNA methylation differences in B cell chronic lymphocytic leukemia
Source: Biol Sex Differ. 2019 Jan 7;10:2. doi: 10.1186/s13293-018-0213-7 (PMC6322323; doi:10.1186/s13293-018-0213-7)
Supplement: Supplementary file 1 — Table S1. Samples for RNA-Seq analysis and replication of DNA methylation. Table S2. CLL subgroups of this study classified by Queiros et al. Table S3. Normal B cells subtypes samples from Kulis et al. Figure S1. Sex-related X chromosomal DMPs stratified by gene features and CpG features. Figure S2a. Thirty-six replicated autosomal DMPs. b. The 7 X chromosomal DMPs of figure 2d in EGA data. c. Genes with numbers of X chromosomal DMPs ≥ 4 in EGA data. Figure S3a. CLL sex-related DEGs. b. The 18 DNAm-DEGs and their DMPs. Figure S4a. There were 549 X chromosomal DMPs with median β < 0.2 or median β > 0.8 in CLL females. b. Genes with at least 3 DMPs in Figure S4a (N=43). Figure S5. MED14 hotspot detected by FEM algorithm. (DOCX 3039 kb) [file 13293_2018_213_MOESM1_ESM.docx]

Sex DNA methylation differences of B cell chronic lymphocytic leukemia

Table S1. Samples for RNA-Seq analysis and replication of DNA methylation.

| Study | CLL Female | CLL Male | Control Female | Control Male |  |
| --- | --- | --- | --- | --- | --- |
| EGAD00010000254 | 51 | 88 | 6 | 8 | 450K DNA  methylation |
| EGAD00010000871 | 65 | 98 | 3 | 4 |  |
|  |  |  |  |  |  |
| EGAD00001000258 | 29 | 58 | 0 | 0 | RNA-Seq |
| GSE66117 | 21 | 26 | 0 | 0 |  |
| GSE16921 | 0 | 0 | 17 | 24 |  |

Table S2. CLL subgroups of this study classified by Querios et al.

|  | U-CLL | I-CLL | M-CLL |
| --- | --- | --- | --- |
| CLL Female | 9 | 5 | 15 |
| CLL Male | 7 | 3 | 9 |

Table S3. Normal B cells subtypes samples from Kulis et al.

|  | CD19+ | CD5+ NBC | NBC | csMBC | ncsMBC |
| --- | --- | --- | --- | --- | --- |
| Female | 6 | 2 | 2 | 2 | 2 |
| Male | 8 | 1 | 1 | 1 | 1 |


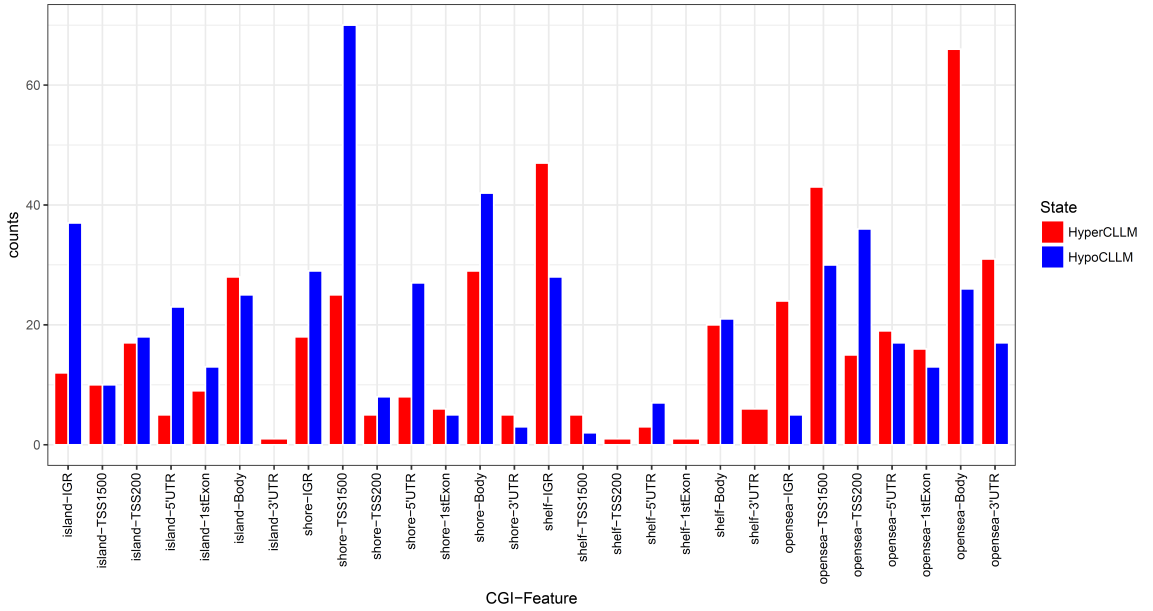


Figure S1. Sex-related X chromosomal DMPs stratified by gene features and CpG features.


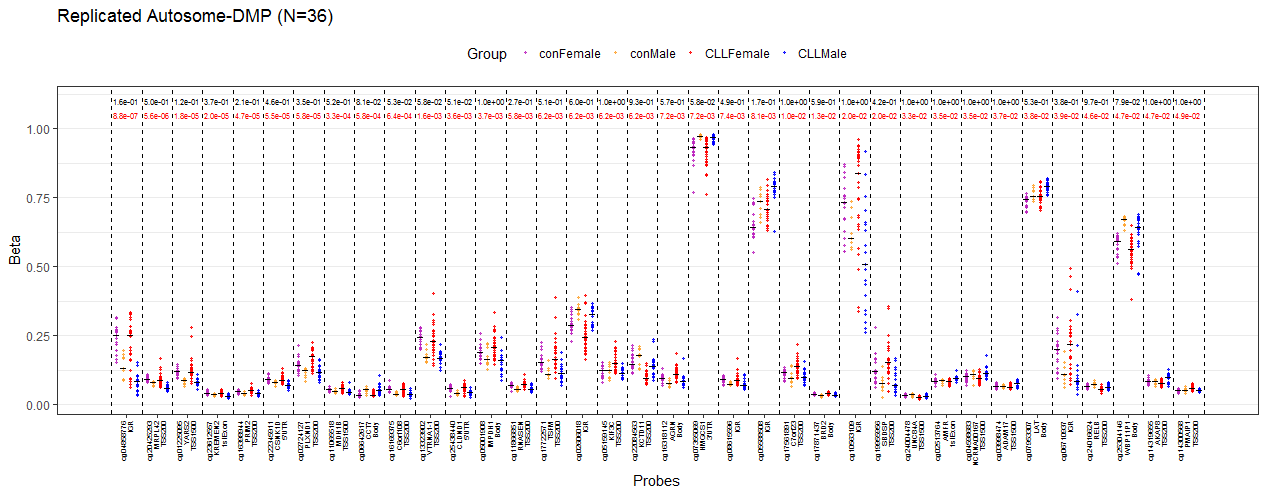


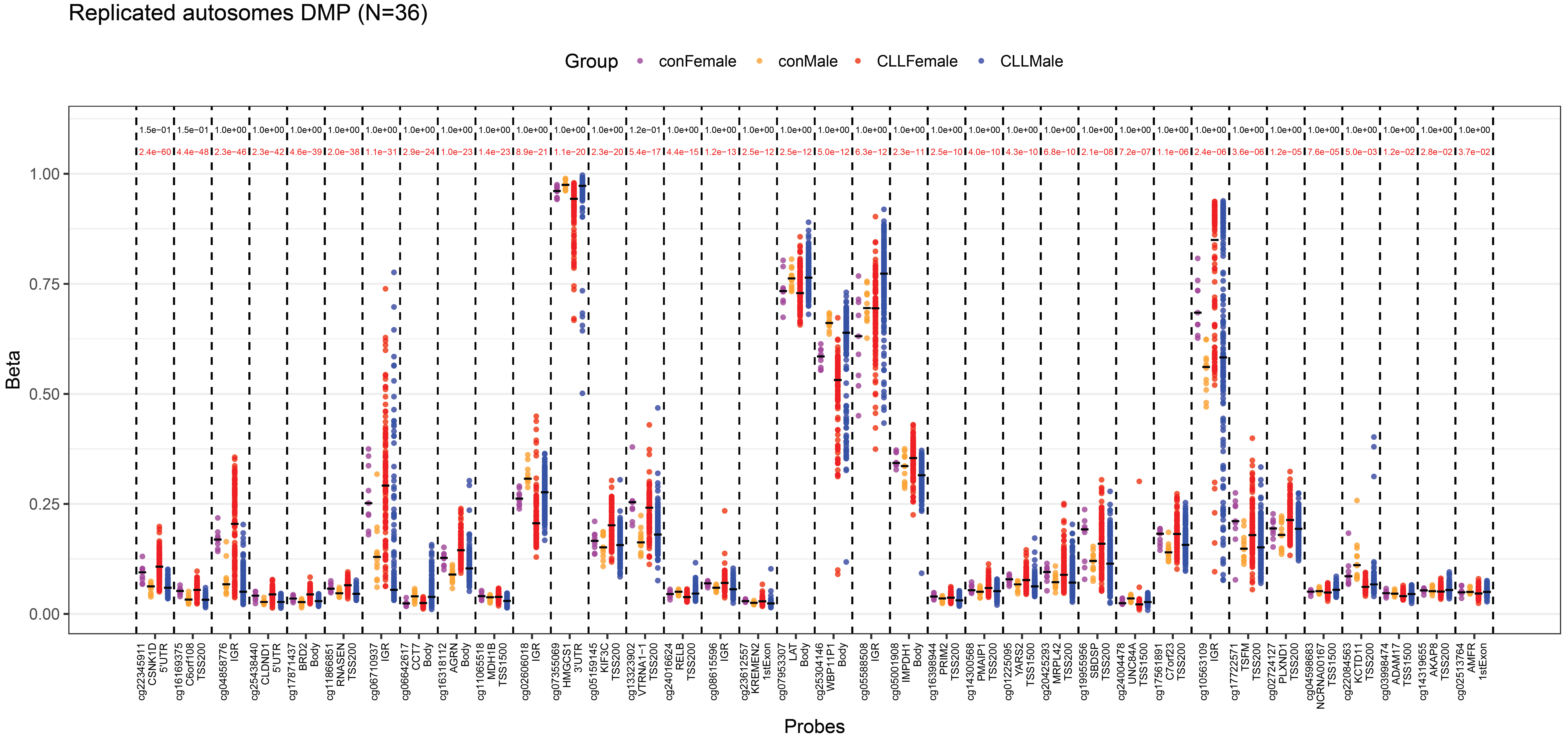


Figure S2a. Thirty-six replicated autosomal DMPs. Upper panel showed the 36 DMPs in our data; lower panel showed the 36 DMPs in EGA data. Black bar indicated medians β value. Q-values were listed at the top, controls were colored in black and CLL were in red.


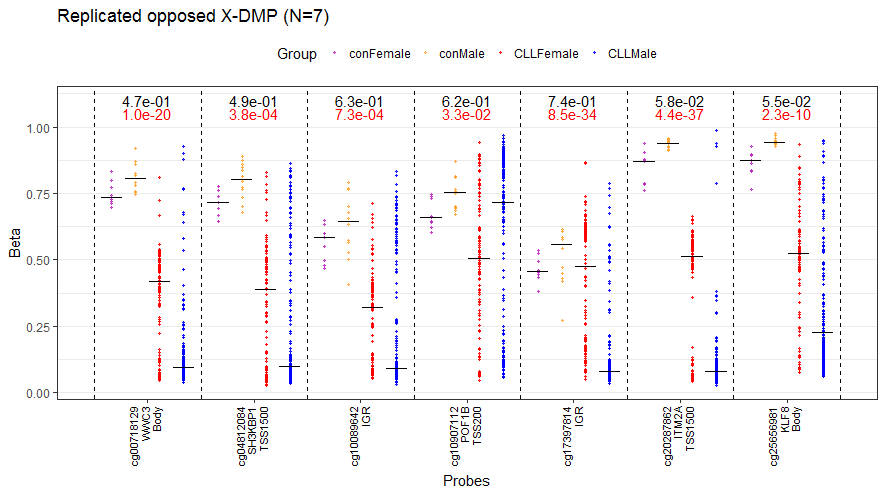


Figure S2b. The 7 X chromosomal DMPs of figure 2d in EGA data.


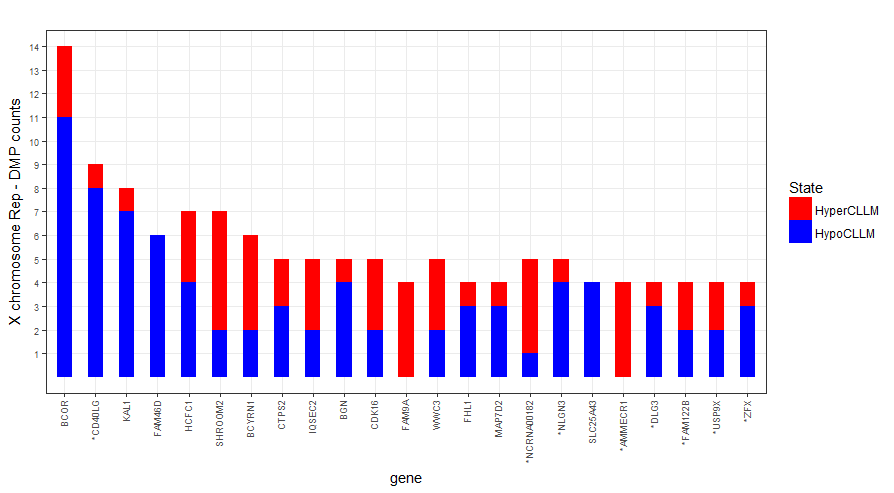


Figure S2c. Genes with numbers of X chromosomal DMPs >= 4 in EGA data. Numbers of DMPs were showed in y-axis. Genes of which all probes could be replicated were marked with “*”.


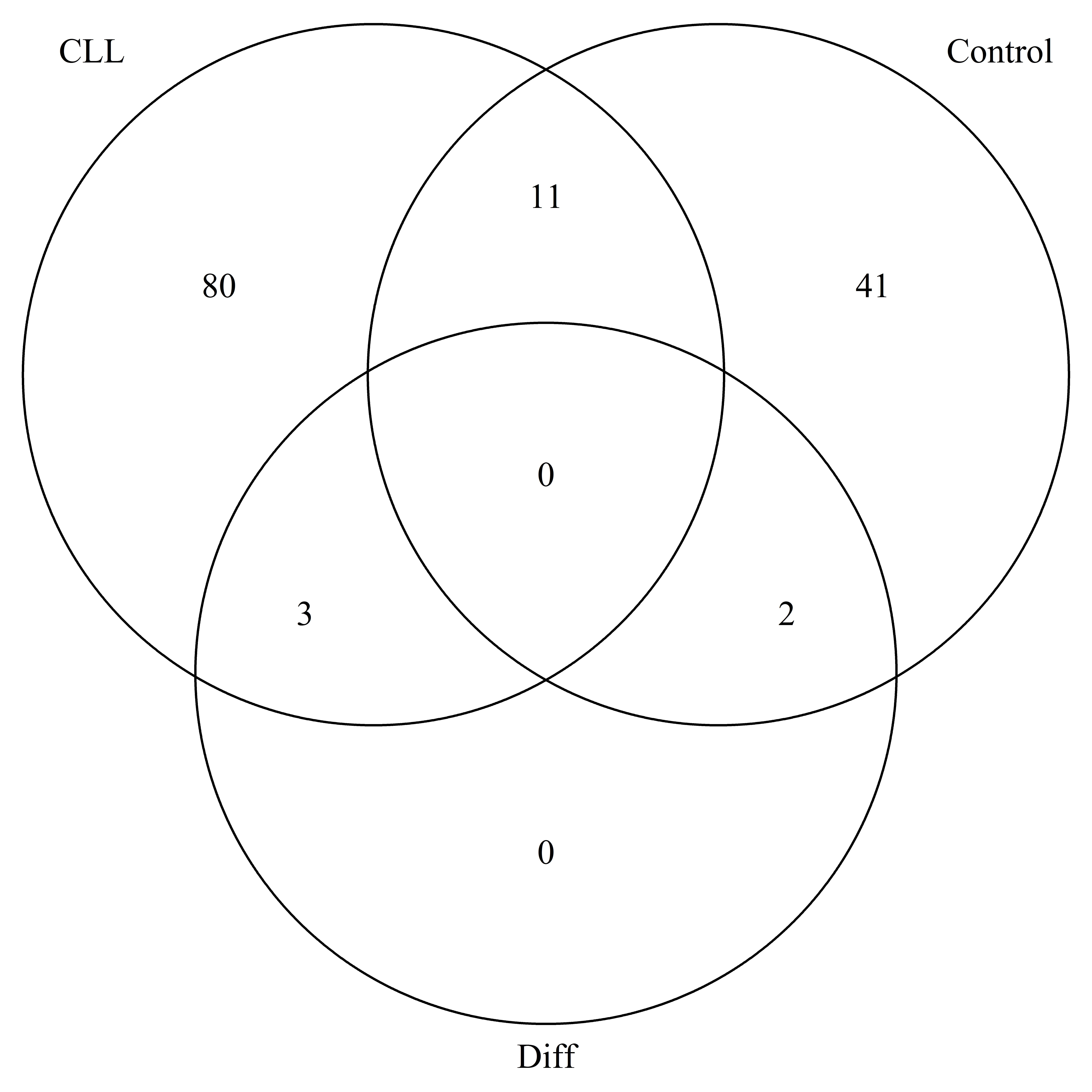


Figure S3a. CLL sex-related DEGs. By pooling the RNA-Seq data, we detected 94 significant genes between sex of CLL, and 54 significant genes between sex of controls, but no significant genes in the interaction term(Diff). The 83 (80+3) genes were considered as DEGs.


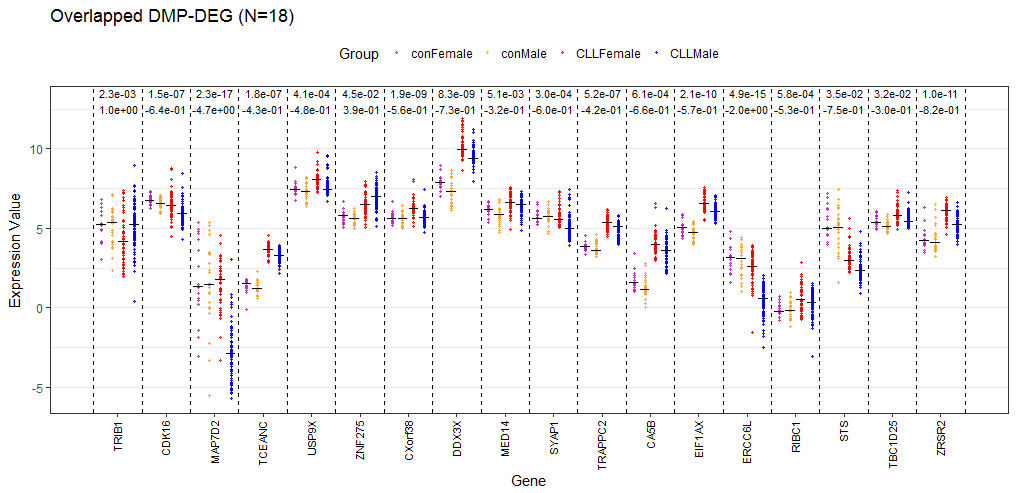


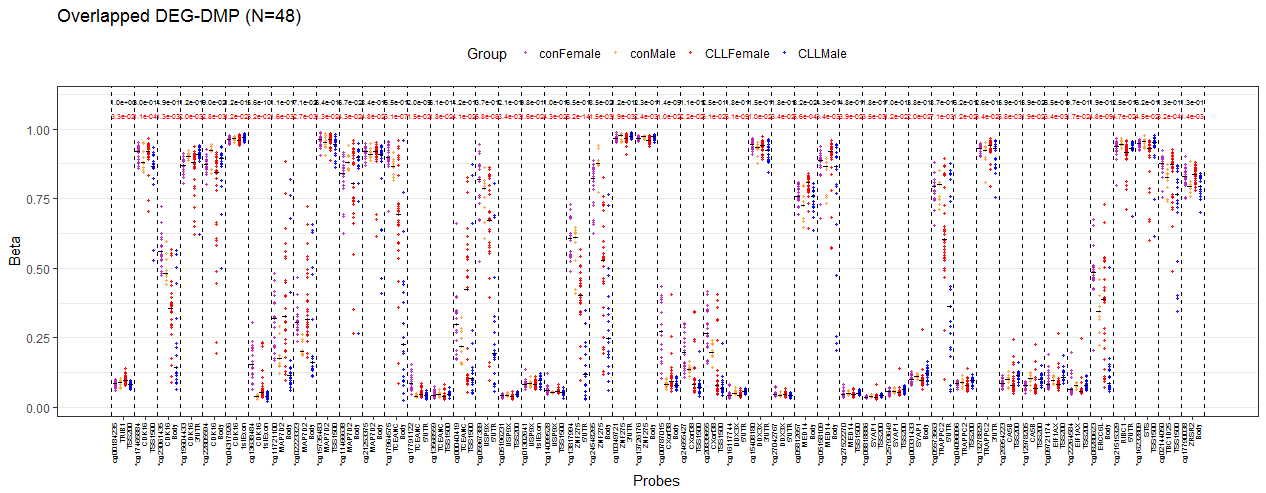


Figure S3b. The 18 DNAm-DEGs and their DMPs. Upper panel, 18 DEGs overlapped with DMPs covered genes. Q-value and log_2_FG were showed in the top of each genes. Lower panel, the 48 DMPs overlapped with DEGs.


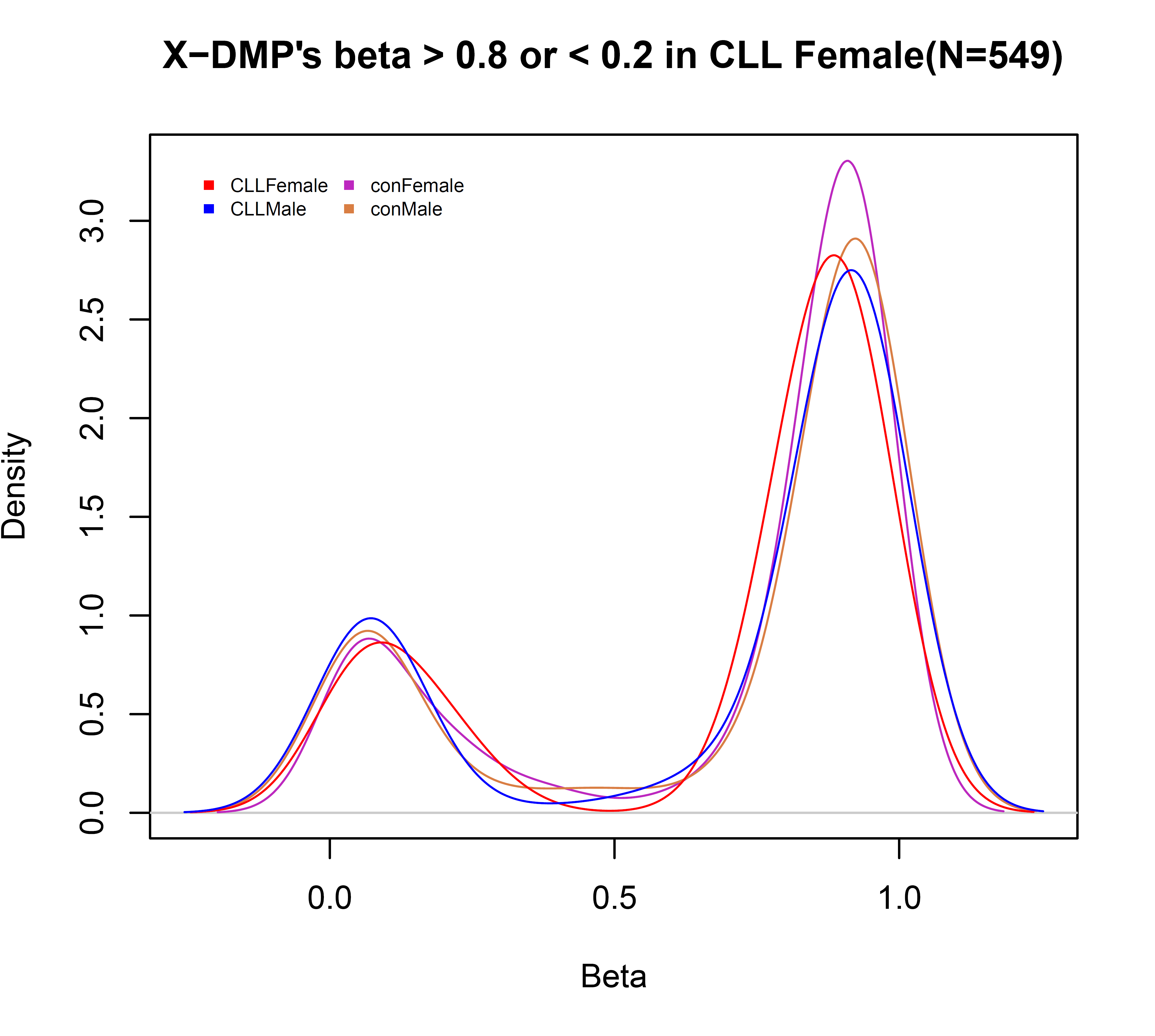


Figure S4a. There were 549 X chromosomal DMPs with median β < 0.2 or median β > 0.8 in CLL females. This figure showed the density of these DMPs’ mean β value.


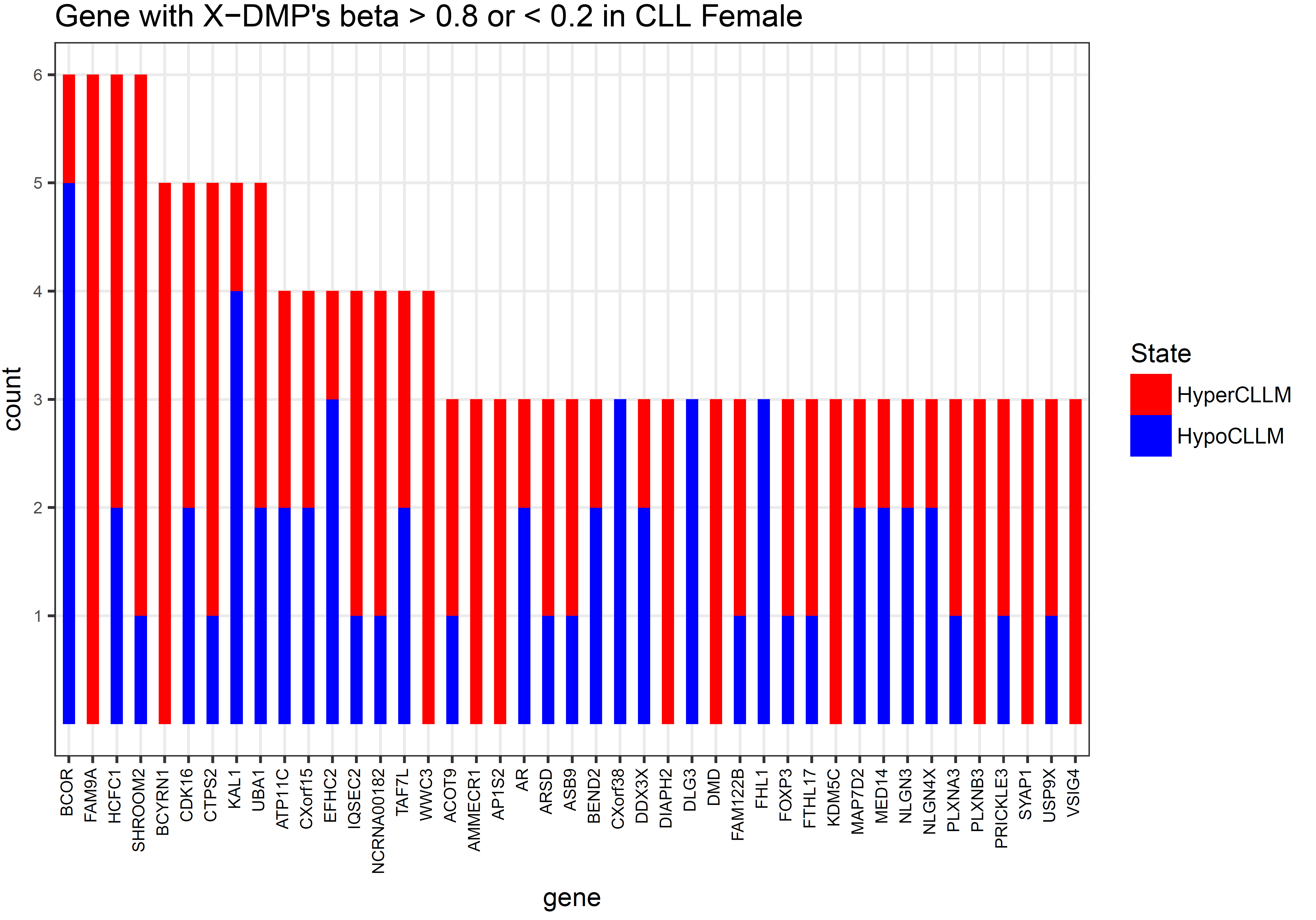


Figure S4b. Genes with at least 3 DMPs in Figure S4a (N=43).


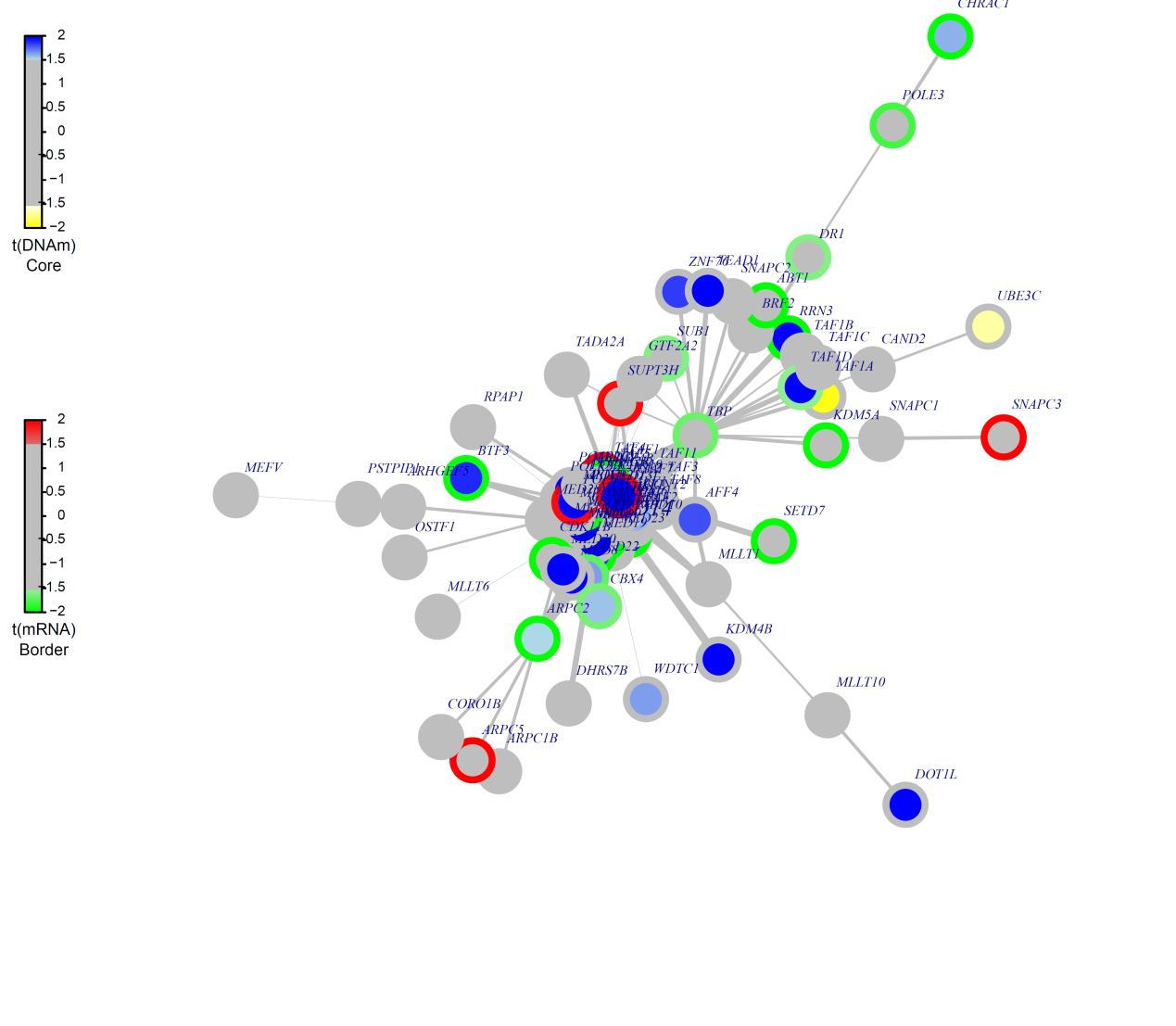


Figure S5. *MED14* hotspot detected by FEM algorithm. T-statistic of DNA methylation was colored in blue or yellow inside the circle, and t- statistic of expression was colored in red or green outside the circle.
