# Supplementary material for: Sex-related DNA methylation differences in B cell chronic lymphocytic leukemia
Source: Biol Sex Differ. 2019 Jan 7;10:2. doi: 10.1186/s13293-018-0213-7 (PMC6322323; doi:10.1186/s13293-018-0213-7)
Supplement: Supplementary file 2 — Supplemental methods. (DOC 93 kb) [file 13293_2018_213_MOESM2_ESM.doc]

**SUPPLEMENTAL MATERIALS AND METHODS**

**450K BeadChip data analysis**

Signal intensities were extracted by the Illumina GenomeStudio software. Samples were removed if more than 1% of CpG sites had detection P > 0.05. CpG sites were removed if more than 1% of samples had detection P > 0.05 and the beadcounts were less than 3 in 5% of samples. Beta values were normalized using quantile normalization plus beta-mixture quantile normalization (QN.BMIQ) [1]. Batch effects were adjusted by ComBat[2]. Sites with annotated SNPs were excluded. Probes in the Y chromosomes and reported to be cross-reactive[3] were also excluded. Altogether, our dataset contains 361,732 autosomal probes, and 9,482 X chromosomal probes from 89 samples. We used the same method for DMP replication datasets, which yielded 363,519 autosomal probes and 9,509 X chromosomal probes.

**Differentially Methylated Region (DMR) analysis**

All DMPs were used to identify DMRs using the R package *DMRcate* with default settings (a DMR must has at least 3 DMPs)[4].

**Gene Ontology (GO) analysis**

Gene ontology term enrichment analysis was performed with the R package *GOstats*[5]. Genes covering the autosomal and X chromosomal DMPs were analyzed separately.

**Functional Epigenetic Module (FEM) analysis**

FEM analysis was performed using the R package *FEM*[6]. FEM seeks modules of functionally related genes that exhibit differential promoter DNA methylation and differential expression by using protein-protein interaction network, assuming an inverse association between promoter DNA methylation and gene expression. Our DNA methylation data and the pooled RNA-Seq data for male and female CLL patients were used as inputs. CpG sites with significant DNA methylation differences between healthy men and women were removed, as well as genes that showed significant expression differences between healthy men and women.

**Details of the interaction term**

We applied the model integrated in the R-bioconductor package *limma* [7] to detected DMP. In this study, we have three comparisons:

1. Which CpGs respond to sex in CLL cases;

model: CpG ~ CLL-M – CLL-F; Referred to “MvsFinCLL” in Fig1a & 2a.

1. Which CpGs respond to sex in controls;

model: CpG ~ Control-M – Ccontrol-F; Referred to “MvsFinCon” in Fig1a & 2a.

1. Which CpGs respond sex differently in CLL compared to control.

model: CpG ~ (CLL-M – CLL-F) – (Control-M – Ccontrol-F); Referred to “Diff” in Fig1a & 2a.

According to the *limma*’s reference manual, model (3) is referred as the *interaction* *term*. It’s a linear model in fact. In other words, we used three models to detect the DMPs (integrated as one in *limma*).

Because the overlapped part of (1) and (2) might still show sex difference, we used (3) to calculate the statistic.

For example, cg05240083 is one of the 39 overlapped sites in figure 2a. It has: Δβ_CLL_ = -0.46, and Δβ_con_ = -0.26. So they both showed significant in model (1) & (2). One can clearly realize that the methylation levels of this site between sexes of CLL differ from control, although they have the same direction. Because we aim to find the sex difference in CLL but not control, it might be incorrect if we removed this site just because (1) and (2) overlap. Model (3) is to resolve this problem. Model (3) indicated that this site had a q-value 1.580148e-07, so we still considered this site as a CLL sex-related DMP. Other examples can go to Figure 2c, these 7 opposite X chromosomal DMPs were defined by Model (3).


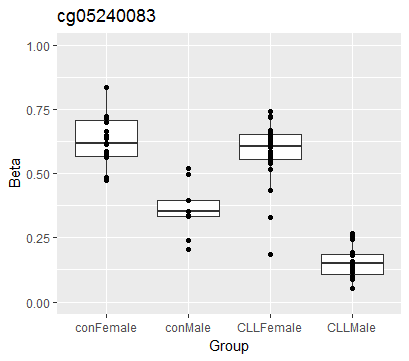


**REFERENCES**

1. Wang T, Guan WH, Lin J, Boutaoui N, Canino G, Luo JH, Celedon JC, Chen W: **A systematic study of normalization methods for Infinium 450K methylation data using whole-genome bisulfite sequencing data**. *Epigenetics* 2015, **10**(7):662-669.

2. Johnson WE, Li C, Rabinovic A: **Adjusting batch effects in microarray expression data using empirical Bayes methods**. *Biostatistics* 2007, **8**(1):118-127.

3. Chen YA, Lemire M, Choufani S, Butcher DT, Grafodatskaya D, Zanke BW, Gallinger S, Hudson TJ, Weksberg R: **Discovery of cross-reactive probes and polymorphic CpGs in the Illumina Infinium HumanMethylation450 microarray**. *Epigenetics* 2013, **8**(2):203-209.

4. Peters TJ, Buckley MJ, Statham AL, Pidsley R, Samaras K, Lord RV, Clark SJ, Molloy PL: **De novo identification of differentially methylated regions in the human genome**. *Epigenet Chromatin* 2015, **8**.

5. Falcon S, Gentleman R: **Using GOstats to test gene lists for GO term association**. *Bioinformatics* 2007, **23**(2):257-258.

6. Jiao YM, Widschwendter M, Teschendorff AE: **A systems-level integrative framework for genome-wide DNA methylation and gene expression data identifies differential gene expression modules under epigenetic control**. *Bioinformatics* 2014, **30**(16):2360-2366.

7. Ritchie ME, Phipson B, Wu D, Hu Y, Law CW, Shi W, Smyth GK: **limma powers differential expression analyses for RNA-sequencing and microarray studies**. *Nucleic Acids Res* 2015, **43**(7):e47.
